# Health-related quality of life in cancer immunotherapy: a systematic perspective, using causal loop diagrams

**DOI:** 10.1007/s11136-022-03110-5

**Published:** 2022-03-17

**Authors:** Elizabeth Beaulieu, Anne Spanjaart, Ashley Roes, Bernard Rachet, Stéphane Dalle, Marie José Kersten, Delphine Maucort-Boulch, Mohammad S. Jalali

**Affiliations:** 1 MGH Institute for Technology Assessment, Harvard Medical School, Boston, MA USA; 2 Department of Hematology, Cancer Center Amsterdam, Amsterdam UMC, University of Amsterdam, Amsterdam, The Netherlands; 3 Department of Non-Communicable Disease Epidemiology, London School of Hygiene & Tropical Medicine, London, UK; 4 Department of Dermatology, Hospices Civils de Lyon, Centre Hospitalier Lyon Sud, Pierre-Bénite, France; 5 Department of Biostatistics, Hospices Civils de Lyon, Lyon, France; 6 Sloan School of Management, Massachusetts Institute of Technology, Cambridge, MA USA

**Keywords:** Immunotherapy, Cancer, Quality of life, Systems science

## Abstract

**Purpose:**

System science offers a unique set of tools, including causal loop diagrams (CLDs), for stakeholders to better grasp the complexity of factors surrounding quality of life. Because the health-related quality of life (HRQoL) of cancer immunotherapy patients exists within an intricate system affected by and affecting many factors across multiple dimensions, the development of a systems-level model can provide a powerful framework to aid the understanding of this complexity. We developed a CLD for HRQoL of cancer immunotherapy patients.

**Methods:**

We first applied a literature-based approach to construct a CLD for patients following immunotherapy. We then iteratively reviewed and enhanced the CLD through interviews with subject matter experts.

**Results:**

Based on the reviewed literature and subject matter expert input, we produced a CLD representing the system surrounding cancer immunotherapy patients’ HRQoL. Several feedback loops are identified that span clinical experiences, oncology teams’ perceptions about immunotherapy, social support structures, and further research and development in cancer immunotherapy, in addition to other components. The CLD enables visualization of thought experiments regarding how a change anywhere in the system can ultimately worsen or improve patients’ HRQoL.

**Conclusion:**

The CLD illustrates the valuable contribution of a systems perspective to quality-of-life research. This systems-based qualitative representation gives insight on strategies to inhibit harmful effects, enhance beneficial effects, and inherent tradeoffs within the system. The CLD identifies gaps in the literature and offers a communication tool for diverse stakeholders. Our research method provides an example for studying the complexities of quality of life in other health domains.

**Supplementary Information:**

The online version contains supplementary material available at 10.1007/s11136-022-03110-5.

## Introduction

System science tools such as causal loop diagrams (CLDs) offer a powerful framework to understand the complex interplay of factors influencing a phenomenon of interest [1]. While they have been widely used in medicine and public health [2–5], their applications in studying quality of life has thus far been limited, e.g., [6]. We demonstrate the usefulness of CLDs in a case study of applying the framework to the system surrounding health-related quality of life in cancer immunotherapy, particularly by integrating findings from prior research with insights from experts. While this report focuses on the case of cancer immunotherapy, it provides an example of expanding the application of CLDs to analyzing the underlying dynamics of quality of life in other health problems. The cancer immunotherapy clinical setting is rapidly evolving and linked with external influences [7]. Strategies to improve outcomes, and which may be mentally modeled using the CLD, include but are not limited to: new treatment options; digital tools; remote monitoring; patients’ choices and contributions; clinical trials; treatment delivered at home; and patients’ advocacy groups.

Immunotherapy is defined generally as medicinal strategies that harness the immune system to prevent or treat disease [8]. Immunotherapy used in the context of modern cancer care has been developed to stimulate and enhance the immune system’s response to tumors [9]. Before the introduction of immunotherapy, options for treating cancer fell into four main categories: radiotherapy, chemotherapy, surgery, and other targeted treatments [9]. Immunotherapy is thus often cited as the fifth pillar of cancer care [10].

Immunotherapy applied as cancer treatment includes the following broad classes: cell-based immunotherapies, immunomodulators, vaccines, antibody-based targeted therapies, and oncolytic viruses [11]. Ipilimumab, an immune checkpoint inhibitor targeting CTLA4 was first approved by European Medicines Agency (EMA) and the U.S. Food and Drug Administration (FDA) in 2011, representing the first major modern immunotherapy treatment for cancer [12, 13]. Immunotherapies for cancer have since received approvals for use in patients with multiple types of cancer. Immunotherapy cancer treatment represents a major innovation in the field of medicine, and has been shown to offer patients favorable outcomes in terms of clinical measures such as overall survival, progression-free survival, and tumor size reduction [14, 15]. Nivolumab and pembrolizumab, anti-programmed death 1 (PD-1) checkpoint inhibitors, were approved by the FDA in 2015. These two checkpoint inhibitors have played a major role in cancer immunotherapy, as they have shown great success in treating melanoma, lymphoma, and non-small cell lung cancer [16] and have demonstrated improvements to health-related quality of life (HRQoL) [17].

Defining health outcomes with purely objective clinical measures such as blood test results, tumor size, and prognosis (i.e., expected survival) overlooks many of the important elements that characterize overall patient well-being. The concept of HRQoL, however, attends to important but non-clinical aspects of health such as social and emotional wellness to measure patients’ overall well-being. A few definitions for HRQoL exist in the literature; the primary one we will employ is “how well a person functions in their life and his or her perceived well-being in physical, mental, and social domains of health” [18]. Accounting for HRQoL is critical in the evaluation of cancer patients’ health because they exhibit many diverse symptoms, including losses of functional ability that are not observable with a laboratory test or imaging procedures [19]. HRQoL outcomes measures are invaluable inputs to guide shared clinical decision-making on choices of therapy [20].

Despite the importance of HRQoL of patients following immunotherapy, it has only received some limited, recent research attention [21, 22]. One noticeable gap is that less HRQoL-focused research has been performed regarding cancer immunotherapy purely due to the treatment’s relative novelty. Research has focused instead primarily on establishing clinical safety and effectiveness, and attention to patient-reported outcomes used to measure HRQoL is under-explored. The current HRQoL research in cancer immunotherapy tends to focus on primary data collection that produces analyses of specific clinical trial observations in terms of a quality-of-life outcome [23], the symptoms and determinants of HRQoL in cancer immunotherapy patients [24], and tools used to measure HRQoL [19], which are actively evolving to suit the specific needs of the population. Further, existing HRQoL instruments were developed mainly for chemotherapy outcomes and not specifically tailored to measure immunotherapy outcomes [25]. Chemotherapy is distinct from immunotherapy as it directly targets tumors, while immunotherapy treats patients by acting on their immune system and can boost the immune response to teach the immune system how to identify and destroy cancer cells. Acute and delayed toxicities dramatically differ between chemo- and immunotherapy [26]. The way that cancer immunotherapy patients’ HRQoL is analyzed in the literature is incomplete in terms of inattention to dynamic interconnections among various individual and social determinants within a broader system. A qualitative systemic understanding can offer practical advantages by generating insight about how factors interconnect and enabling proactive, impactful decision-making.

To address this research gap, we set an objective to develop a CLD that identifies the system surrounding this patient group’s HRQoL qualitatively by mapping out factors that interact around HRQoL following cancer immunotherapy.

## Methods

The research method consists of: (1) a review of the literature to develop the initial CLD, and (2) interviews with clinical experts to enhance and finalize the CLD. These two steps are elaborated below.

A semi-systematic review approach was employed to gather variables related to HRQoL and their potential interactions. A search in PubMed in August of 2020 applied the query (((immunotherapy[Title/Abstract]) OR ("cancer treatment"[Title/Abstract])) OR ("cancer patient*"[Title/Abstract])) AND ("quality of life"[Title/Abstract]). The search strategy was intentionally broad to capture any research that focused on quality of life with respect to cancer treatment. Results were sorted by most to least relevant and research assistants independently checked article titles and abstracts to verify overall relevancy and collected variables connected to cancer patients’ quality of life from the articles’ full text. The research assistants are undergraduate students who are knowledgeable in the fundamentals of public health and system dynamics. Initial findings were reviewed by a third researcher to calibrate understanding before proceeding with the variable extraction process. We identified potential factors relevant to HRQoL until we observed a saturation point of factors already extracted, such that continuing to extract information from the sorted list of studies was no longer producing unique new variables. Then, we explored the literature-based evidence supporting causal relationships.

Once we had constructed an internal first draft of the CLD based upon literature, we conducted semi-structured interviews with eight subject matter experts knowledgeable in cancer immunotherapy treatment. All experts were medical doctors and their expertise spans a wide range of topics, including but not limited to epidemiology, gastrointestinal oncology, melanoma, hemato-oncology, CART-cell therapy, and immune checkpoint inhibitors. Countries represented by experts include France, Netherlands, Portugal, Spain, U.K., and U.S. In each interview, we presented the CLD, explained the meaning of each variable, and walked through each feedback loop step by step. We then asked if the representation correlated with their experience and understanding of the system, whether any variable represented was not relevant, and if any relevant variables were absent. Finally, we asked if the links (arrows) and polarities (± signs on the arrows) were consistent with their belief about causality. From this structured point, the conversations would vary depending on the feedback and we would ask follow-up questions depending on specific points brought up by the expert. We applied an iterative approach by implementing feedback to the CLD draft after each interview and presenting the revised version to the next expert. The CLD version following the final interview was returned to the interviewees for additional feedback. These interviews provide essential inputs in our analysis because: (1) individual studies might not take a broad and systematic perspective and thus study only a subset of factors or connections among factors; (2) limited research in this space may overlook important factors. The CLD is thus reflective of evidence found in the literature as well as critical inputs from all eight experts interviewed.

The CLD includes reinforcing (R) and balancing (B) feedback loops. In a reinforcing feedback loop, an increase (or decrease) in one variable feeds through effects in other variables represented in the loop, and through those mechanisms ends up increasing (or decreasing) the initial variable even more. Meanwhile in a balancing loop, an increase (or decrease) in one variable produces feedback effects which decrease (or increase) the starting variable. In other words, reinforcing loops cause changes in the same direction as the initial variable change direction, and balancing loops cause changes in the inverse direction as the initial variable change direction.

## Results

Forty articles were reviewed. The extracted factors that potentially influence HRQoL are reported in Table 1. Three articles ([27–29]) contributed an initial basis of many factors and additional articles often supported relevance of the same variables. Additional factors identified through interviews are also reported in Table 1.

**Table 1:** Variables of the causal loop diagram. Factors extracted from the literature listed in the second column correspond to aggregated causal loop diagram variables listed in the first column

	Causal Loop Diagram Variable	Extracted Factor in Quality of Life System
**Individual Patient Level**		
	Physical wellbeing	Physical function [28, 30]; role function [21, 27, 31]; activity [28]; appearance [28]; self-care [32]; debilitation [28]; diminished strength [30]; extent to which can perform usual household activities/usual work [32]; fatigue [30, 32]; functional impairment [33]; mobility/physical activity [32]; sexual function [28, 34]; sleeplessness [32]; comorbidities [35]; health status [27]; organ function [34]
	Psychological wellbeing	Mental function [28]; negative emotions [30]; cognitive disorder [32]; cognitive function [21]; body image [36]; emotional function [21, 28, 31, 36]; emotional stress [37]; malaise/depression [32]; psychosocial stress [36]; psychological well-being [35]; psychosocial function [34]; psychosocial status [38]; habitual optimism [39]; life stress [35]; day-to-day stress [27]; anxiety [32]; sense of meaning [27]
	Patients’ willingness to undergo immunotherapy	Self-selection to treatment [40]; patient attitudes and perceptions [41]
	Receipt of immunotherapy	Treatment [28, 35, 42]
	Effectiveness of immunotherapy treatment	Clinical outcome [28]; immunotherapeutic agents [43]
	Expected survivorship	Overall survival [27, 38, 44–46]; recurrence-free survival [44]; progression-free survival [45]
	Negative side effects due to immunotherapy	Pain [30, 32, 47]; treatment complications/side effects other than pain [32, 38, 44, 48]; relative importance of symptoms [32]; with or without complications during treatment [47]
	Work/leisure activities	Ability to work [49, 50]; occupational performance [31]; work status [28]; purpose/personal goals [28]
	Affordability of treatment	Family income per capita [47]; financial status [28]; resources [27, 28]; socioeconomic status (SES) – education/income [35]; earnings [51]; patient or payer cost [44]; affordability [46]; health care resource use [33]
	Accessibility of treatment	Health care access barriers [52, 53]
	Oncology team size/presence of multidisciplinary knowledge network	Same variable^expert opinion^
	Receipt of support	Social support [35]; support [27]; interconnectedness [27]; social function [31]; social health [36]
	Social connection	Social isolation [28]
	Individual stigma about asking for support	Experiences of stigma by cancer patients [54]
	Communication of need for social support	Experiences of stigma by cancer patients [54]
	Individual optimism/grit	Same variable^expert opinion^
	Disappointment from previous treatments	Same variable^expert opinion^
**Immediate Connections Level**		
	Oncology team’s likelihood to recommend immunotherapy	Clinician or family/friend treatment recommendation [44]
	Effective management of side effects by health care system	Same variable^expert opinion^
	Quantity and quality of social support structure	Living situation [27]; neighborhood [27]; network [28]; caregiver presence [28]; caregiver's prognostic awareness [55]; family [28]; availability of help [28]
**Greater Community Level**		
	Incentives to invest in immunotherapy R&D	Drivers of innovation [46, 56]; R&D spending [57]; strategies to develop precision medicine [47]
	Favorable clinical trial results	Same variable^expert opinion^
	Level of evidence for immunotherapy	Same variable^expert opinion^
	Medical guidelines in favor of immunotherapy	Cancer drug regulatory approvals [58]; medical society statements [59]
	Availability of treatment	Same variable^expert opinion^
	Oncology team’s perceived effectiveness	Same variable^expert opinion^
	Patient education that encourages them to reframe perspectives and overcome individual stigma	Informal support groups [60]; interventional efforts to alleviate stigma [61]
	Availability of resources for social support e.g., patient support groups	Ready availability of advice and material help [28]
	Social stigma associated with, and experienced by, cancer patients	Cancer stigmatization within healthy population [62]

### Feedback loops

**Fig. 1:**
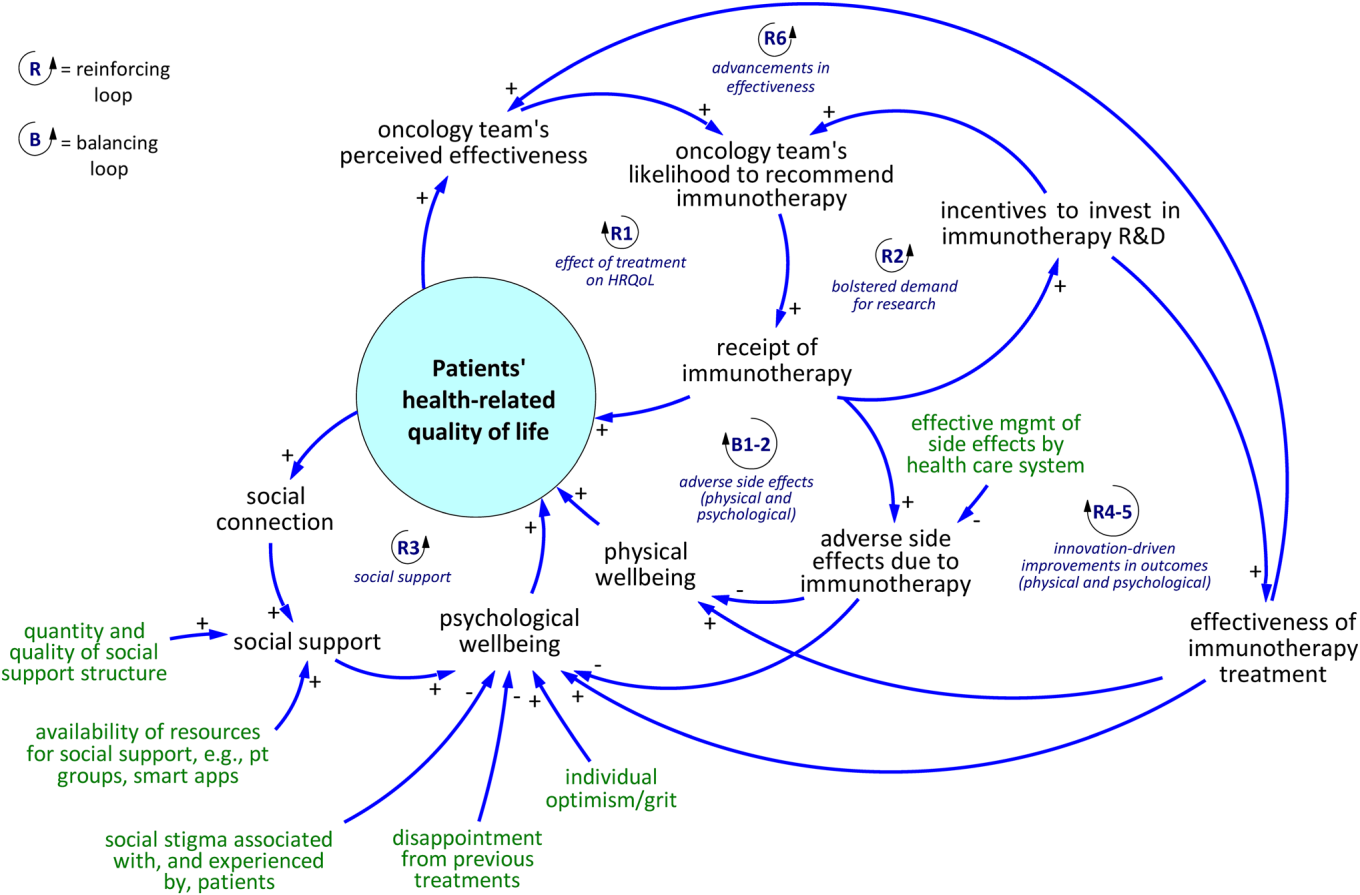
Simplified Causal Loop Diagram. The extended version showing all variables is presented in the appendix

We highlight six reinforcing feedback loops and two balancing feedback loops in Fig. 1. We describe these feedback effects briefly below.

#### Reinforcing loop R1

Consider reinforcing loop R1. The *oncology team’s perceived effectiveness* represents the clinicians’ overall impression about the quality of treatment and confidence that the treatment option is the option most likely to produce clinical success for their patients. Perceived effectiveness directly impacts *oncology team’s likelihood to recommend immunotherapy*. Recommendation by the patient’s immediate team is a foremost deciding factor in the patients’ decision to undergo the treatment and results in *receipt of immunotherapy*. As shown in effectiveness studies, patients’ HRQoL improves as a result of the receipt of immunotherapy such that this central variable increases in the responding patients [63]. In aggregate over time, patients’ positive experience in terms of improved HRQoL flows up to community-level perceptions about the treatment thus elevates again the first variable: *oncology team’s perceived effectiveness*.

#### Reinforcing loop R2

Reinforcing loop R2 begins with a change in the volume of patients receiving immunotherapy (*receipt of immunotherapy*). For example, this might increase due to new favorable clinical evidence. The increase signals the market and research community to focus resources on research and development activities in cancer immunotherapy, offering further data and refining evidence that helps build confidence in oncologists’ treatment decisions.

#### Reinforcing loop R3

Another reinforcing mechanism relates to the link between patients’ HRQoL and social aspects of health. Consider when HRQoL decreases due to, for instance, adverse side effects occurring during treatment. *Social connection* then decreases due to factors such as feeling of alienation (i.e., a shift in identify as a cancer patient as being different and separate from the healthy population that can lead to self-isolation) and a decreased ability to engage in normal work and leisure activities. When *social connection* decreases, *social support* may also decrease by way of stigma experienced by patients against communicating the need for support. Decreased *social support* begets lower *psychological well-being* and thus an overall decrease in HRQoL. See the appendix figure for a more detailed view of the mechanisms in this loop.

#### Reinforcing loops R4 and R5

To understand reinforcing loops R4 and R5, consider an increase in *incentives to invest in immunotherapy R&D*. Over time, investment in research activities produce improvements in the *effectiveness of immunotherapy treatment*, leading to improved *physical well-being* (via R4) and *psychological well-being* (via R5) for immunotherapy patients. When either or both aspects of well-being increase, so does *patients’ health-related quality of life.* The remaining pieces of the loop—*oncology team’s perceived effectiveness* through *incentives to invest in immunotherapy R&D*—have already been described above within loops R1 and R2.

#### Reinforcing loop R6

R6 illustrates how advances in *effectiveness of immunotherapy treatment* produced by research activities (*incentives to invest in immunotherapy R&D*) directly affects the oncology team’s perceived effectiveness of the treatment. Again, the remaining pieces of the loop (*oncology team’s perceived effectiveness* through *incentives to invest in immunotherapy R&D*) have been described above. The link between effectiveness and perceived effectiveness represents an independent reinforcing feedback effect.

#### Balancing loops B1 and B2

Within balancing loops B1 and B2, *receipt of immunotherapy* often results in *adverse side effects due to immunotherapy*. Adverse side effects have detrimental effects on both *physical well-being* (B1) and *psychological well-being (B2)* which negatively impact the central variable *patients’ health-related quality of life*. When the central variable decreases, *oncology team’s perceived effectiveness* also decreases, as does the *oncology team’s likelihood to recommend immunotherapy*. Lower likelihood for the oncologists to recommend immunotherapy to patients decreases *receipt of immunotherapy.*

## Discussion

The CLD provides a systematic perspective and offers an aid to explain phenomena that have the potential to bolster or deteriorate HRQoL. The literature and expert-based approach allowed identification of a complex interplay of factors with multiple feedback mechanisms present in the system. A wide set of variables was identified. Mechanisms at play in the system ranged from factors at the individual patient level up to dynamics operating at the greater community level. The expanded version of the diagram, shown in the supplement as Figure S-1, delineates these levels and presents a discussion of their components.

The manner that component parts relate to one another and not only how they act separately produces the overall system behavior. A shift within just one feedback loop has the potential to alter the resulting levels of variables throughout the system. Feedback mechanisms thus act as important leverage points. We use the diagram to detect patterns in behavior within this system and identify opportunities to disrupt harmful feedback mechanisms that reduce patients’ HRQoL, strengthen feedback mechanisms that operate to improve patients’ HRQoL, and understand systemic tradeoffs.

### Disrupting harmful effects

Consider first feedback mechanisms that have potentially severe detrimental effects if left unchecked. R3 in Fig. 1 shows an example. For HRQoL to thrive in the system, *psychological well-being* must be upheld. *Social support* is a direct determinant of *psychological well-being*, which is in jeopardy of deteriorating precipitously should *social connection* drop to activate a negative reinforcing mechanism. The loop can produce a downward spiral and severely worsen HRQoL. The strength of exogenous variables influencing R3 component variables such as *quality and quantity of social support structures* and *availability of resources for social support e.g., patient support groups/smart apps* serve as proactive protectors inhibitive of the potentially harmful effects.

COVID-19 effects are not represented in the diagram, as it was designed as a generic representation not specific to particular years. However, the pandemic is relevant to mention as it pertains to the dynamics discussed above. Cancer patients, in particular patients receiving some types of immunotherapy such as CD19-directed chimeric antigen receptor T-cells [64, 65], are highly vulnerable to SARS-CoV-2 infection and development of severe symptoms [66]. They have thus been socially distancing in extreme measure in recent years, making the social support structures in place for immunotherapy patients all the more important in light of the dynamics highlighted by the CLD.

### Enhancing beneficial effects

Here we identify a beneficial feedback cycle scenario. Notice the R1 and R2 loops in Fig. 1, which consist of components described in the above section. Patients’ *receipt of immunotherapy* connects to the variable for investment in research and development. The research activities produce evidence that flows into *oncology team’s likelihood to recommend immunotherapy* and back into the individual patient level (*receipt of immunotherapy*) which then bolsters HRQoL.

The implication is a need to enable amplification of this cycle while maintaining integrity in the evidence upon which decisions are based. Opportunities to amplify R2 include direct investment in research by public and/or private sectors and timely inclusion of evidence-based findings pertaining to immunotherapy treatments where appropriate.

### Awareness of tradeoffs

When *receipt of immunotherapy* increases, the change creates opposing effects within the system via balancing loops (B1 and B2) and reinforcing loops (R1 and R2). The dueling effects triggered by an increase in *receipt of immunotherapy* highlights a tradeoff within the system. More patients receiving immunotherapy will at once activate mechanisms that would push it in the direction of increasing even more through the reinforcing loops. At the same time, it also activates a balancing effect that pulls *receipt of immunotherapy* downward. The dynamics are described in greater detail in the appendix.

The effects that will win over directionally in terms of the net effect on receipt of immunotherapy and ultimately on patients’ health-related quality of life depend on the systemic timing and strength of flows between variables. Circumstances that produce a net positive effect cannot be answered with certainty in our qualitative representation. The tradeoff dynamics noticeable in the CLD would be a direction for investigation of a future quantified model. However, in the CLD we can still hypothesize strategies to mitigate the potentially harmful balancing loop effects and stack the deck in favor of a net positive effect on HRQoL. One such strategy would be strengthening the immediate connections level variable *effective management of side effects by health care system*. Managing side effects better has the potential to interrupt B1 by mitigating the harmful effects from treatment side effects on *physical well-being, psychological well-being,* and *patients’ health-related quality of life*. In addition to management, it is also equally important for health care systems to better scrutinize and report potential side effects.

### Limitations

Among limitations to this study, we identify the two we consider most critical in the context of HRQoL systems science. First, the process of constructing the CLD has been dependent on limited published research of HRQoL in patients receiving the relatively novel treatment of cancer immunotherapy. Current research on this topic is active but does not yet fully characterize all aspects of the CLD. Second, the CLD is limited by the perspectives of eight subject matter experts. While each interview was extensive and detailed, and experts’ feedback contributed insight from a diverse set of experiences related to cancer immunotherapy treatment, time and availability constraints limited in the number of interview subjects.

Despite these limitations, the qualitative CLD helps highlight the complexity of the systems of various factors that may affect and be affected by HRQoL following immunotherapy. The flow of relationships among factors allows visualization of feedback mechanisms that are intuitive when laid out in the diagram yet not obvious when thinking about many factors abstractly, i.e., without a visual aid. Additionally, the CLD offers a powerful communication tool that allows expansion of the scope of consideration to a diverse set of policymakers and stakeholders involved in immunotherapy care. It can act as a vehicle to simplify understanding of the complexity so that separate actors, who are sometimes confined to just one part of the problem, can better collaborate to achieve the common goal of improved outcomes.

This work might be used in the future to create quantified simulation models. Such models would then estimate and project the impact of interventions on specific outcomes of interest. They would also enable comparisons of outcomes resulting from a portfolio of interventions and allow decision-makers to optimally allocate resources. Availability of data will determine how the development of such models can proceed. Changes in HRQoL can be quantified using patient-reported outcomes (PRO) instruments. PRO instruments frequently used in cancer research include the generic EQ-5D and the cancer-specific EORTC QLQ-C30 questionnaires [67]. Novel, immunotherapy-specific instruments are also an area of active development among ongoing efforts continue to collect HRQoL data from patients, including in our large study in several European countries; see the project website for more information [68].

## Supplementary Information

Below is the link to the electronic supplementary material.

Supplementary file1 (PDF 294 kb)
