# Supplementary material for: Health-related quality of life in cancer immunotherapy: a systematic perspective, using causal loop diagrams
Source: Qual Life Res. 2022 Mar 17;31(8):2357–66. doi: 10.1007/s11136-022-03110-5 (PMC8929267; doi:10.1007/s11136-022-03110-5)
Supplement: Supplementary file 1 — Supplementary file1 (PDF 294 kb) [file 11136_2022_3110_MOESM1_ESM.pdf]

Supplementary Material for

Health-Related Quality of Life in Cancer Immunotherapy: A Systematic Perspective, Using Causal Loop Diagrams

Three primary levels of variables

The development of the CLD resulted in a diagram with three interrelated tiers separated based upon the primary level of the variables: individual patient, immediate connections, and greater community. To highlight an example, essential medical factors are experienced by the patient directly within the pathways to receiving immunotherapy and include clinical effects stemming from the treatment itself. The results of the treatment aggregate up to broader perceptions about immunotherapy and also affect the economics of future research and development in this area. Separate physical and psychological pieces that feed into patients' HRQoL each contain important linkages that contribute to determining the central HRQoL variable. The CLD thus displays the variety of variables in the system and their interconnectedness. Supplementary Figure S-1 displays the full version of the CLD with all variables separated into these three levels. A simplified version of the figure is shown in Figure S-1.

At the individual patient level, core components of clinical factors surrounding HRQoL in patients following immunotherapy, physical and psychological components of HRQoL, and the effectiveness of the treatment. The individual patient layer of the CLD represents a wide range of variables and mechanisms, from the central variable patients' health-related quality of life (HRQoL); receipt of immunotherapy treatment and its affordability, accessibility, and patients' willingness to undergo it; associated clinical factors such as survivorship and experience of adverse side effects; psychological and physical components of HRQoL. The central box shows interconnections at this variable level.

Important medical factors central to the treatment receipt and its connection to HRQoL are part of the individual patient layer. Patients' willingness to undergo the treatment positively affects the likelihood of actualizing receipt of immunotherapy. As shown by clinical effectiveness studies, receipt of immunotherapy improves HRQoL as well as expected survivorship. The treatment can also cause patients to experience adverse side effects, however, which decreases HRQoL via physical wellbeing. These medical components at the individual patient level play important roles in feedback loops that are completed at higher layers of the CLD and will be described in detail later in Results.

Flowing from the individual-level variables, the next section of the diagram concerns variables related to not just individual patients but patients' immediate connections such as their respective partner, friends, and family. The second layer in the appendix figure shows that this level contributes variables that represent potentially important agents to impact dynamics within the patient level and ultimately patients' HRQoL. Three variables make up this level: *quality and quantity of social support structure*, *effective management of side effects by health care system*, and *oncology team's likelihood to recommend immunotherapy*.

The level above the patients' immediate connections contains variables representative of the greater community, including community resources and economic drivers for innovation. These

are shown in the outermost, third layer in the appendix figure. The addition of this third section to the aforementioned individual-patient- and immediate-connections-level components completes the CLD. Community-level variables include indicators of the overall environment in terms of social support and educational resources, the degree of social stigma within the cultural climate, availability of the treatment, investment in further research and development which feeds into medical guidelines and the oncology team's perceived effectiveness of immunotherapy. This perceived effectiveness variable is a key link to complete feedback loops.

As shown in Figure S-1's innermost layer, four feedback loops exist using exclusively individual-level variables to complete the loops. This set of reinforcing loops correspond to the reinforcing loop labeled R3 in the simplified version of the figure shown in the main article text (i.e., Figure 1). They include: R3-A (the degree of social connection's inverse relationships with individual stigma); R3-B (similarly, HRQoL's effect on ability to connect with others socially and the link with psychological wellbeing); R3-C (individual stigma's ability to decrease individual outreach for social support and actualized receipt of that support); and R3-D (HRQoL affecting the ability to engage in everyday work and leisure activities and psychological wellbeing). We describe two of these feedback loops within the individual-level variables in more detail below.

Reinforcing Loop R3-D: R3-D is a reinforcing loop consisting of three variables. Improvement of the central variable *patients' health-related quality of life* improves *work/leisure activities*.[1-4]

When patients are able to actively engage in their day-to-day professional and recreational activities to a greater degree, their *psychological wellbeing* increases as result.[5-7] Increased *psychological wellbeing* links back to improve overall HRQoL[5] which completes the reinforcing loop. An initial increase in HRQoL becomes elevated to an even greater degree via the R3-D loop components.

Reinforcing Loop R3-C: R3-C is another reinforcing loop within individual-level variables and concerns patients' wellbeing with respect to their propensity to connect socially. We can start this loop with variable *social connection.* A drop in social connection is frequently experienced by cancer patients generally.[8] This phenomenon can be attributed to a sense of alienation from previous social identity or feeling "othered" by negative connotations associated with cancer.[9] When *social connection* decreases, perhaps due to a difficult time period during immunotherapy treatment, *individual stigma regarding asking for support* can increase as the patient becomes more and more isolated and averse to reaching out for help.[10] When *individual stigma regarding asking for support* increases, the likelihood that the patient will ask for social support decreases (*communication of need for social support*),[11] as does the *receipt of support* resultantly.[11] Patients' decrease in support being received completes the loop and *social connection* decreases further.[7]

Variables existing primarily on other levels such as *Quality and quantity of social support structure* (immediate-connections level) and *patient education to overcome individual stigma* (community-level) affect individual-level variables in R3-C. These exemplify how variables at those other tiers can play important roles to impact individual-level feedback dynamics affecting patients' HRQoL.

Details on Tradeoffs

The balancing loop B1 highlighted in the CLD along with the dynamics in reinforcing loop R1 helps to demonstrate trade-offs inherent in the system. The important variable to focus on to begin the discussion about trade-offs is *receipt of immunotherapy*. When patients receive immunotherapy, individual patients in aggregate sends signals to the greater community level

variables (1) *oncology team's perceived effectiveness*, via *patients' health-related quality of life*, and (2) *incentives to invest in immunotherapy R&D*. The former activates reinforcing loops R1 and R2, which ultimately act to increase *receipt of immunotherapy* to an even greater degree, holding everything else constant. The latter activates reinforcing loop R2 which also acts to increase again the starting variable *receipt of immunotherapy*. *Receipt of immunotherapy* directly improves the central variable of interest, *patients' health-related quality of life.* Therefore, we can see that an increase in patients receiving immunotherapy activates two reinforcing feedback mechanisms.

However, *receipt of immunotherapy* also (3) activates balancing loops B1 and B2, which have a balancing effect: as more patients receive immunotherapy, more side effects are experienced in aggregate by the patient population. These adverse effects can weaken *physical wellbeing* and *psychological wellbeing* and consequently *patients' health-related quality of life* to reduce oncologists' perceived effectiveness as well as the recommendation of the treatment by oncologists. Patients' *receipt of immunotherapy* thus decreases. Through the balancing loop, an initial increase in *receipt of immunotherapy* moves through the loop to cause treatment receipt to decrease rather than increase.

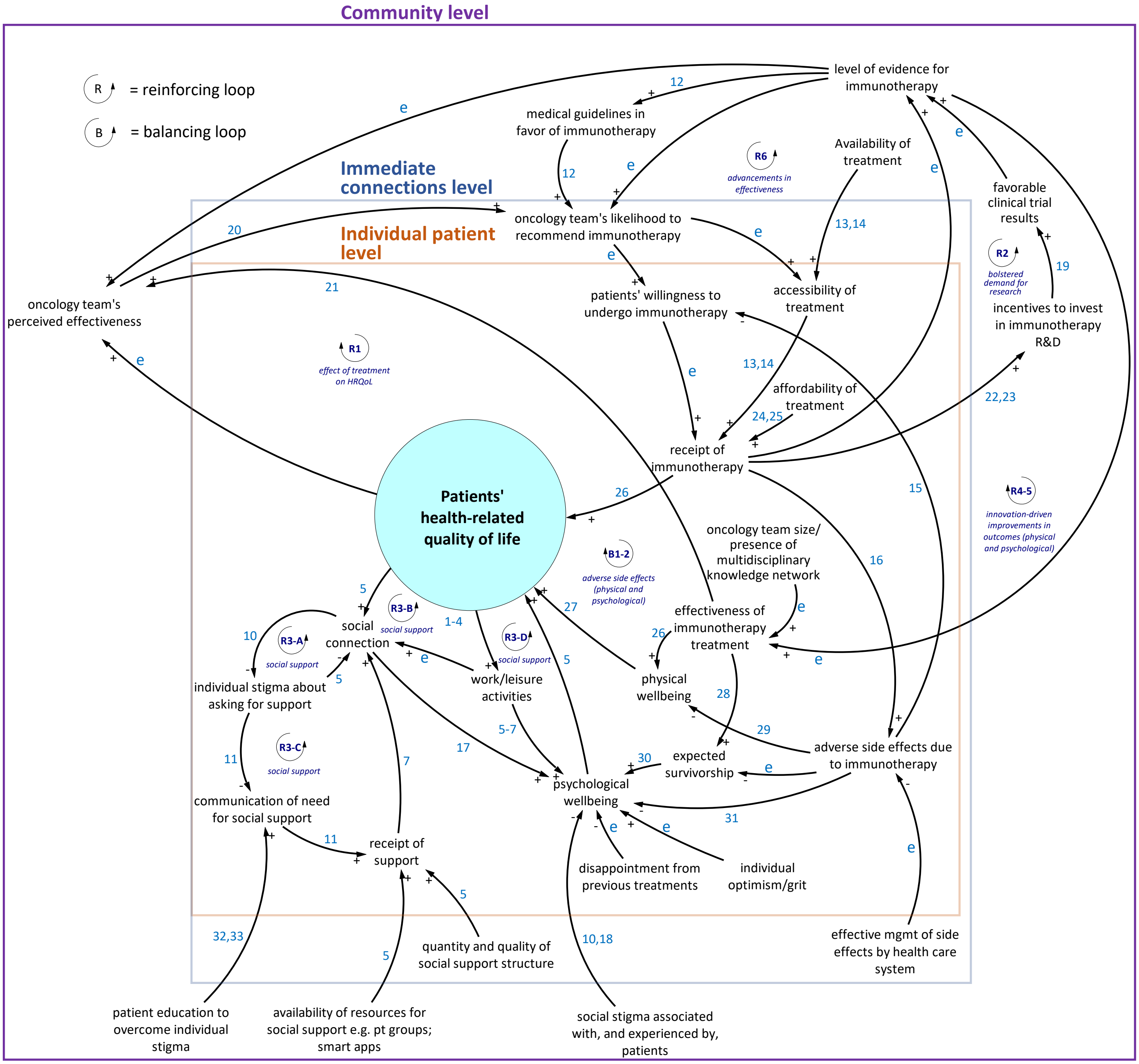

Figure S-1: Expanded Causal Loop Diagram. This expanded view shows addition detail of identified variables and the primary level of each variable: individual-patient-, immediate-connection-, and community-level. The numbers on causal links presents references; "e" represents extracted links from interviews with experts.

References:

1. Henry DH, Viswanathan HN, Elkin EP, Traina S, Wade S, Cella D. Symptoms and treatment burden associated with cancer treatment: results from a cross-sectional national survey in the U.S. *Supportive Care in Cancer* 2008; **16**(7): 791-801.
2. Baxi SS, Salz T, Xiao H, et al. Employment and return to work following chemoradiation in patient with HPV-related oropharyngeal cancer. *Cancers of the Head & Neck* 2016; **1**(1): 4.
3. Bradley CJ, Oberst K, Schenk M. Absenteeism from work: the experience of employed breast and prostate cancer patients in the months following diagnosis. *Psycho-Oncology* 2006; **15**(8): 739-47.
4. Feuerstein M, Todd BL, Moskowitz MC, et al. Work in cancer survivors: a model for practice and research. *Journal of Cancer Survivorship* 2010; **4**(4): 415-37.
5. Donovan K, Sanson-Fisher RW, Redman S. Measuring quality of life in cancer patients. *Journal of Clinical Oncology* 1989; **7**(7): 959-68.
6. Wells M, Williams B, Firnigl D, et al. Supporting 'work-related goals' rather than 'return to work' after cancer? A systematic review and meta-synthesis of 25 qualitative studies. *Psycho-Oncology* 2013; **22**(6): 1208-19.
7. Kagawa-Singer M, Padilla GV, Ashing-Giwa K. Health-related quality of life and culture. *Semin Oncol Nurs* 2010; **26**(1): 59-67.
8. Davis H, Vetere F, Ashkanasy S, et al. Towards social connection for young people with cancer. Proceedings of the 20th Australasian Conference on Computer-Human Interaction: Designing for Habitus and Habitat. Cairns, Australia: Association for Computing Machinery; 2008. p. 319–22.
9. Adams RN, Mosher CE, Abonour R, Robertson MJ, Champion VL, Kroenke K. Cognitive and Situational Precipitants of Loneliness Among Patients With Cancer: A Qualitative Analysis. *Oncol Nurs Forum* 2016; **43**(2): 156-63.
10. Yılmaz M, Dissiz G, Usluoğlu AK, Iriz S, Demir F, Alacacioglu A. Cancer-Related Stigma and Depression in Cancer Patients in A Middle-Income Country. *Asia Pac J Oncol Nurs* 2019; **7**(1): 95-102.
11. Knapp S, Marziliano A, Moyer A. Identity threat and stigma in cancer patients. *Health Psychol Open* 2014; **1**(1): 2055102914552281-.
12. Cohen EEW, Bell RB, Bifulco CB, et al. The Society for Immunotherapy of Cancer consensus statement on immunotherapy for the treatment of squamous cell carcinoma of the head and neck (HNSCC). *Journal for ImmunoTherapy of Cancer* 2019; **7**(1): 184.
13. Karanth S, Fowler ME, Mao X, et al. Race, Socioeconomic Status, and Health-Care Access Disparities in Ovarian Cancer Treatment and Mortality: Systematic Review and Meta-Analysis. *JNCI Cancer Spectrum* 2019; **3**(4).
14. Lin Y, Wimberly MC, Da Rosa P, Hoover J, Athas WF. Geographic access to radiation therapy facilities and disparities of early-stage breast cancer treatment. *Geospatial Health* 2018; **13**(1).
15. Islam KM, Anggondowati T, Deviany PE, et al. Patient preferences of chemotherapy treatment options and tolerance of chemotherapy side effects in advanced stage lung cancer. *BMC Cancer* 2019; **19**(1): 835.
16. Winer A, Bodor JN, Borghaei H. Identifying and managing the adverse effects of immune checkpoint blockade. *J Thorac Dis* 2018; **10**(Suppl 3): S480-S9.
17. Ashing-Giwa KT. The contextual model of HRQoL: a paradigm for expanding the HRQoL framework. *Qual Life Res* 2005; **14**(2): 297-307.

18. Vrinten C, Gallagher A, Waller J, Marlow LAV. Cancer stigma and cancer screening attendance: a population based survey in England. *BMC Cancer* 2019; **19**(1): 566.
19. Huber MA, Kraut N. Key drivers of biomedical innovation in cancer drug discovery. *EMBO Mol Med* 2015; **7**(1): 12-6.
20. Steensma DP, Komrokji RS, Stone RM, et al. Disparity in perceptions of disease characteristics, treatment effectiveness, and factors influencing treatment adherence between physicians and patients with myelodysplastic syndromes. *Cancer* 2014; **120**(11): 1670-6.
21. Mellstedt H, Gaudernack G, Gerritsen WR, et al. Awareness and understanding of cancer immunotherapy in Europe. *Hum Vaccin Immunother* 2014; **10**(7): 1828-35.
22. Carrera PM, Kantarjian HM, Blinder VS. The financial burden and distress of patients with cancer: Understanding and stepping-up action on the financial toxicity of cancer treatment. *CA: A Cancer Journal for Clinicians* 2018; **68**(2): 153-65.
23. Prasad V, Mailankody S. Research and Development Spending to Bring a Single Cancer Drug to Market and Revenues After Approval. *JAMA Internal Medicine* 2017; **177**(11): 1569-75.
24. Marlow NM, Pavluck AL, Bian J, Ward EM, Halpern MT. The Relationship Between Insurance Coverage and Cancer Care: A Literature Synthesis. Research Triangle Park (NC): RTI Press; 2009.
25. Altice CK, Banegas MP, Tucker-Seeley RD, Yabroff KR. Financial Hardships Experienced by Cancer Survivors: A Systematic Review. *J Natl Cancer Inst* 2017; **109**(2).
26. Laetsch TW, Myers GD, Baruchel A, et al. Patient-reported quality of life after tisagenlecleucel infusion in children and young adults with relapsed or refractory B-cell acute lymphoblastic leukaemia: a global, single-arm, phase 2 trial. *Lancet Oncol* 2019; **20**(12): 1710-8.
27. Ashing-Giwa KT, Lim J-W. Predicting Health-related Quality of Life: Testing the Contextual Model Using Structural Equation Modeling. *Applied Research in Quality of Life* 2008; **3**(3): 215-30.
28. Song P, Yang D, Wang H, et al. Relationship between the efficacy of immunotherapy and characteristics of specific tumor mutation genes in non-small cell lung cancer patients. *Thorac Cancer* 2020; **11**(6): 1647-54.
29. Faury S, Foucaud J. Health-related quality of life in cancer patients treated with immune checkpoint inhibitors: A systematic review on reporting of methods in randomized controlled trials. *PLoS One* 2020; **15**(1): e0227344-e.
30. Philip EJ, Merluzzi TV, Zhang Z, Heitzmann CA. Depression and cancer survivorship: importance of coping self-efficacy in post-treatment survivors. *Psycho-Oncology* 2013; **22**(5): 987-94.
31. Zhou Y, Cartmel B, Gottlieb L, et al. Randomized Trial of Exercise on Quality of Life in Women With Ovarian Cancer: Women's Activity and Lifestyle Study in Connecticut (WALC). *JNCI: Journal of the National Cancer Institute* 2017; **109**(12).
32. Egestad H. The significance of fellow patients for head and neck cancer patients in the radiation treatment period. *Eur J Oncol Nurs* 2013; **17**(5): 618-24.
33. Hamann HA, Ver Hoeve ES, Carter-Harris L, Studts JL, Ostroff JS. Multilevel Opportunities to Address Lung Cancer Stigma across the Cancer Control Continuum. *J Thorac Oncol* 2018; **13**(8): 1062-75.
